# Supplementary material for: Cell-type specific expression of oncogenic and tumor suppressive microRNAs in the human prostate and prostate cancer
Source: Sci Rep. 2018 May 8;8:7189. doi: 10.1038/s41598-018-25320-z (PMC5940660; doi:10.1038/s41598-018-25320-z)
Supplement: Supplementary file 1 — Supplementary Information [file 41598_2018_25320_MOESM1_ESM.pdf]

Supplementary Information

Cell-type specific expression of oncogenic and tumor suppressive microRNAs in the human prostate and prostate cancer

Binod Kumar, Avi Z. Rosenberg, Su Mi Choi, Karen Fox-Talbot, Angelo M. De Marzo, Larisa Nonn, W. Nathaniel Brennen, Luigi Marchionni, Marc K. Halushka, Shawn E. Lupold

Supplementary Table S1-S2

Supplementary Figure S1-S3

Supplementary Table S1. Characteristics of University of Washington and Johns Hopkins radical prostatectomy specimens applied to Figure 1

A) University of Washington Primary Prostate Cancer Samples (Figure 1A)

Sample ID	Tissue Type	Primary Gleason	Secondary Gleason	Tertiary Gleason	Total Gleason
00-074B	primary prostate cancer	3	4	0	7
02-022B	primary prostate cancer	3	4	0	7
04-051A	primary prostate cancer	3	4	0	7
04-055B	primary prostate cancer	3	4	0	7
04-106D	primary prostate cancer	3	3	0	6
05-056C	primary prostate cancer	3	4	0	7
05-120A	primary prostate cancer	3	4	0	7
05-131B	primary prostate cancer	3	4	0	7
05-186B	primary prostate cancer	3	4	0	7
07-094B	primary prostate cancer	3	4	0	7
07-100A	primary prostate cancer	4	3	5	7
09-017B	primary prostate cancer	4	3	0	7
09-049C	primary prostate cancer	4	3	0	7
09-118D	primary prostate cancer	4	3	0	7
10-058A	primary prostate cancer	4	5	0	9

B) JHU Samples (Figure 1B)

Subject	Age	Tissue Type	Gleason Grade
1	60	primary prostate cancer	3+4
2	68	primary prostate cancer	3+4
3	58	primary prostate cancer	3+4
4	65	primary prostate cancer	4+3

Supplementary Table S2. Sequence Read Archive Samples and Categorization

<u>Adrenal</u>
Adrenal SRR944040
Adrenal SRR944031
Adrenal SRR944034

<u>Bladder</u>
Bladder epithelium DRR013035
Bladder Epithelium DRR013036
Bladder DRR013040
Bladder DRR013041
Bladder DRR013042
Bladder DRR013043
Bladder DRR013044
Bladder Epithelium DRR013038
Bladder Epithelium DRR013037
Bladder SRR333658
Bladder SRR333660
Bladder SRR333662
Bladder SRR333664
Bladder SRR333666
Bladder SRR333668
Bladder SRR333670
Bladder SRR333672
Bladder SRR333674

<u>Blood</u>
Plasma SRR822438
Blood SRR1949839
Blood SRR1949840
Blood SRR1949841
Blood SRR1949842
Blood SRR1949843
Blood SRR1949844

Blood SRR1949845
Blood SRR1949846
Blood SRR1949847
Blood SRR1949848
Blood SRR1949849
Blood SRR2061740
Blood SRR2061741
Blood SRR2061742
Blood SRR2061743
Blood SRR2061744

Blood SRR2061745
Blood SRR2061746
Blood SRR2061747
Blood SRR2061748
Blood SRR2061749
Blood SRR2201546
Blood SRR2201547
Blood SRR2201548
Blood SRR2201549
Blood SRR2201550
Blood SRR2201551
Blood SRR2201572
Blood SRR2201573
Blood SRR837471
Blood SRR837472
Blood SRR837473
Blood SRR837474
Blood SRR837475
Blood SRR837476
Blood SRR837477
PBMC SRR926589
Plasma Cell SRR060984
Plasma SRR822437
Plasma SRR822439
Plasma SRR822443
Plasma SRR822444
Plasma SRR822445
Plasma SRR822446
Plasma SRR822450
Plasma SRR822451
Platelet ERR747965
Platelet ERR747966
Platelet ERR747967
Platelet SRR1592244
Platelet SRR1592245
Platelet SRR1592246
Platelet SRR1592247
Platelet SRR1592248
Platelet SRR1592249
RBC SRR1664893
RBC SRR1664894
RBC SRR1664895
RBC SRR1664896
RBC SRR1664897

Brain

Cerebrum SRR1635904
Prefrontal Cortex SRR1635903
Brain ERR038405
Brain ERR038406
Brain ERR038407
Brain ERR038408
Brain ERR038409
Brain, Frontal SRR448330
Brain, Frontal SRR448331
Cerebellum SRR553573
Frontal cortex SRR553572
H9_Diff_ForeBrain SRR1988291
H9_Diff_ForeBrain SRR1988292
H9_Diff_ForeBrain SRR1988293
H9_Diff_ForeBrain SRR1988294
H9_Diff_MidBrain SRR1988299
H9_Diff_MidBrain SRR1988300
H9_Diff_MidBrain SRR1988301
H9_Diff_MidBrain SRR1988302
Prefrontal Cortex ERR409878
Prefrontal Cortex ERR409879
Prefrontal Cortex ERR409882
Prefrontal Cortex ERR409883
Prefrontal Cortex ERR409887
Prefrontal Cortex ERR409888
Prefrontal Cortex ERR409891
Prefrontal Cortex ERR409893
Prefrontal Cortex ERR409896
Prefrontal Cortex ERR409898
Prefrontal Cortex ERR409900
Prefrontal Cortex SRR1759212
Prefrontal Cortex SRR1759213
Prefrontal Cortex SRR1759214
Prefrontal Cortex SRR1759215
Prefrontal Cortex SRR1759216
Prefrontal Cortex SRR1759217
Prefrontal Cortex SRR1759218
Prefrontal Cortex SRR1759219
Prefrontal Cortex SRR1759220

<u>Cervix</u>
Cervix SRR040010
Cervix SRR040012
Cervix SRR040014
Cervix SRR040018
Cervix SRR040024

Colon
Colon SRR649562
Colon SRR837819
Colon SRR837824
Colon SRR837827
Colon SRR837830
Colon SRR837833
Colon SRR837836
Colon SRR837839
Colon SRR837842

Eye
Retina ERR973601
Retina ERR973602
Retina ERR973603
Retina ERR973604
Retina ERR973605
Retina ERR973606
Retina ERR973607
Retina ERR973608
Retina ERR973611
Retina ERR973612
Retina ERR973613
Retina ERR973614
Retina ERR973615
Retina ERR973616
Retina ERR973617
Retina ERR973618

Fat
Fat SRR772343
Fat SRR772344
Fat SRR772345
Fat SRR772353
Fat SRR772354
Fat SRR772356
Fat SRR772359
Fat SRR772360
Fat SRR772362
Fat SRR772363
Fat SRR772366
Fat SRR772367
Fat SRR772368
Fat SRR772369
Fat SRR772370

Fibroblast
Fibroblast_lung SRR1988283
Fibroblast_lung SRR1988284
Fibroblast_lung SRR1988285
Fibroblast_lung SRR1988286

Heart
Heart SRR1635905
Heart ERR038425
Heart ERR038426
Heart ERR038427
Heart SRR553574

Immune
B cell Centroblast SRR060981
B Cell Centrocyte SRR060982
B Cell_CD77 SRR1209512
B Cell_CD77 SRR1209513
B Cell_CD77 SRR1209514
B Cell_CD77 SRR1209515
B Cell_memory SRR060986
B Cell_naive SRR060985
B Cell_Pre-germinal center SRR060983
CD14+ monocytes SRR527681
CD4 T Cell SRR1532971
CD4 T Cell SRR1532972
CD4 T Cell SRR1532973
CD8+ T Cell SRR1853806
CD8+ T Cell SRR1853807
CD8+ T Cell SRR1853808
CD8+ T Cell SRR1853809
Dendritic SRR1708906
Dendritic SRR1708907
Dendritic SRR1708908
Dendritic SRR1708909
Dendritic SRR1708910
Dendritic SRR1708911
Dendritic SRR1708912
Dendritic SRR1708913
Dendritic SRR1708914
Dendritic SRR1708915
Dendritic SRR1708916
Dendritic SRR1708917
Dendritic SRR1708918
Dendritic SRR1708919

Dendritic SRR1708920
Dendritic SRR1708921
Dendritic SRR1708922
Dendritic SRR1708923
Macrophage ERR356515
Macrophage ERR356519
Macrophage ERR356520
Macrophage ERR356521
Macrophage SRR1532974
Macrophage SRR1532975

Kidney
Kidney SRR1635906
Kidney ERR038420
Kidney ERR038421
Kidney ERR038422
Kidney ERR038423
Kidney ERR038424
Kidney SRR553575

Liver
Liver SRR950887
Liver SRR950886
Liver ERR038410
Liver ERR038411
Liver ERR038412
Liver ERR038413
Liver ERR038414

Lung
Lung SRR372642
Lung SRR1635907
Lung SRR1216774
Lung SRR1216779
Lung SRR1216789
Lung SRR1216801
Lung SRR1216823
Lung SRR1216836
Lung SRR1216838
Lung SRR1216840
Lung SRR1608271
Lung SRR1608272
Lung SRR1608273
Lung SRR1608274
Lung SRR1608275
Lung SRR1608276

Lung SRR1608277
Lung SRR1608278
Lung SRR1608279
Lung SRR1608280
Lung SRR1608281
Lung SRR1608282
Lung SRR1608283
Lung SRR1608284
Lung SRR1608285
Lung SRR372614
Lung SRR372616
Lung SRR372618
Lung SRR372620
Lung SRR372624
Lung SRR372626
Lung SRR372630
Lung SRR372632
Lung SRR372634
Lung SRR372636
Lung SRR372638
Lung SRR372640
Lung SRR372642
Lung SRR372644
Lung SRR372646
Lung SRR372648
Lung SRR372650

Pancreas
Pancreas ERR852089
Pancreas ERR852091
Pancreas ERR852092
Pancreas ERR852097
Pancreas ERR852099

Placenta
Placenta SRR567635
Placenta SRR567636
Placenta SRR567637
Placenta SRR567638
Placenta SRR567642
Placenta SRR567643
Placenta SRR567644
Placenta SRR567645
Placenta SRR822452
Placenta SRR822453
Placenta SRR822454

Placenta SRR822455
Placenta SRR822456
Placenta SRR822457
Placenta SRR822458
Placenta SRR822459

Prostate
Prostate_SRR1051368
Prostate_SRR1051375
Prostate_ SRR1051385
Prostate_SRR1051377
Prostate_SRR1051376

Skeletal Muscle
Skeletal muscle SRR1820680
Skeletal Muscle SRR1635908

Skin
Epidermis SRR1259212
Dermis SRR1259216
Dermis SRR1259218
Dermis SRR1259220
Dermis SRR1259222
Dermis SRR1259224
Epididymis SRR384894
Skin SRR1259226
Skin SRR1259228
Skin SRR1259230
Skin SRR1259236
Skin SRR2174541
Skin SRR2174542
Skin_ SRR2174509
Skin_ SRR2174513
Skin_ SRR2174517
Skin_ SRR2174521
Skin_ SRR2174525
Skin_ SRR2174529
Skin_ SRR2174533

Testes
Testes ERR038415
Testes ERR038416
Testes ERR038417
Testes ERR038419
Testes SRR333676
Testes SRR333678

Testes SRR333680
Testes SRR333682
Testes SRR333684
Testes SRR333686
Testes SRR333688
Testes SRR553576

Thyroid
Thyroid SRR1291267
Thyroid SRR1291268
Thyroid SRR1291269
Thyroid SRR1658401
Thyroid SRR1658402
Thyroid SRR1658403
Thyroid SRR1658404
Thyroid SRR1658405
Thyroid SRR1658406
Thyroid SRR1658407
Thyroid SRR1658408

Uterus
Uterus SRR1556139
Uterus SRR1556141
Uterus SRR1556143

Supplementary Fig.S1

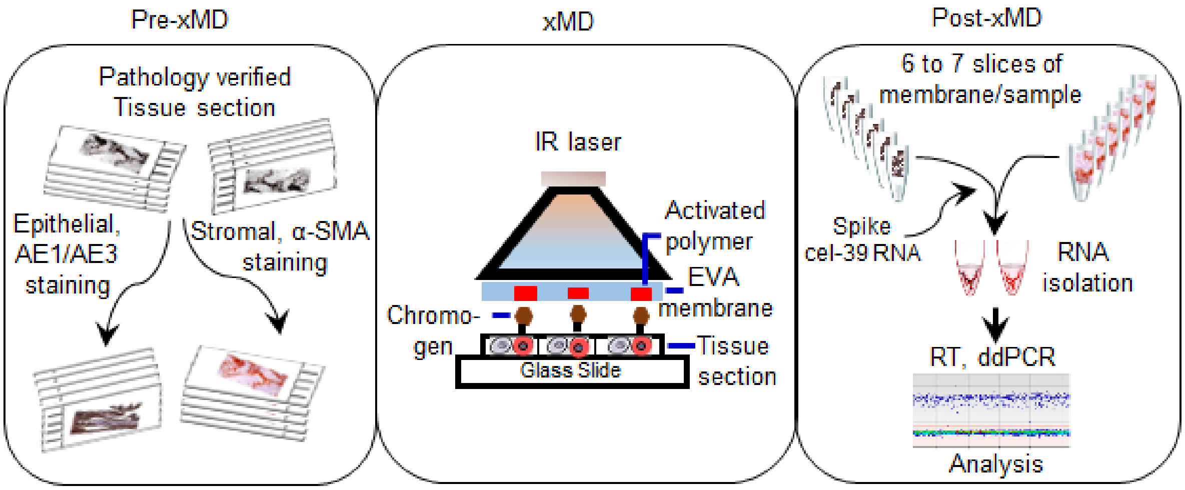

Supplementary Figure S1. Schematic of xMD isolation of prostate stroma and epithelium. FFPE slides (n=6-7 per sample) were either scraped for macrodissection analyses or IHC stained for stroma (anti-α-SMA) or epithelium (anti-AE1/AE3). The region of interest was overlaid with EVA membrane, sealed, and exposed 5 times at power 5 to a flash lamp. The membrane was removed, further dissected by light microscopy and stored at -80°C. The resulting membranes were combined for each sample and total RNA extracted. Internal control cel-39 was added and Total RNA extracted. MicroRNAs were quantified by RT-ddPCR.

Supplementary Figure S2.

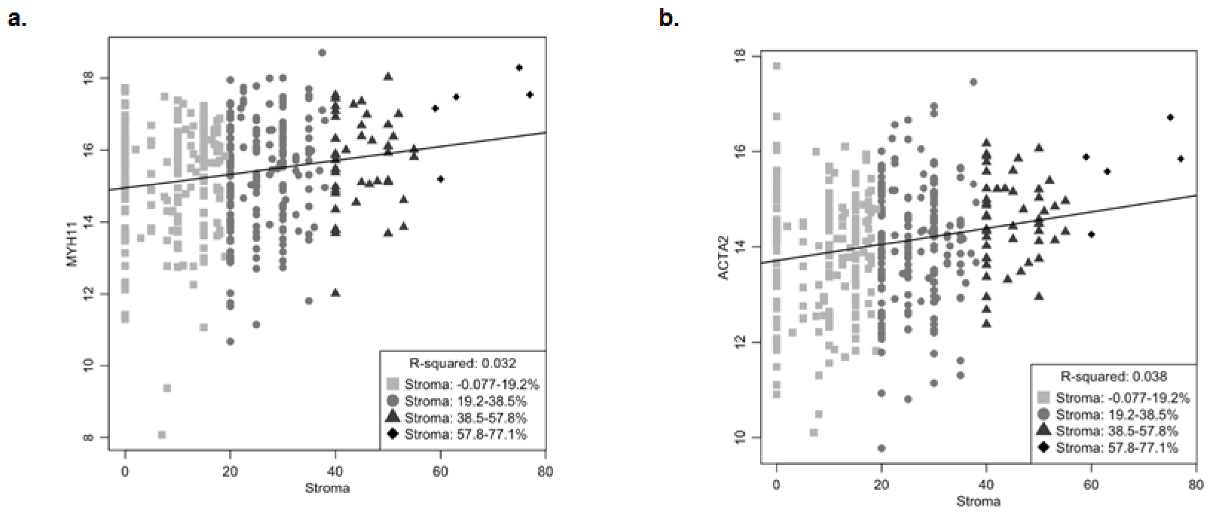

Supplementary Figure S2. Correlation of TCGA-reported stromal cell content and stromal-specific marker expression. **a**, Weak positive association of Smooth Muscle Myosin Heavy Chain 11 (MYH11) mRNA level and reported stromal percentages. p-value: 1.7 × $^{10-4}$. **b**, Weak positive association of Smooth Muscle Actin (ACAT2) mRNA level and reported stromal percentages. p-value: 4.8×10^{-5}. Pearson's correlation coefficient determined by linear regression analysis and fitted using least-squares method.

Supplementary Figure S3

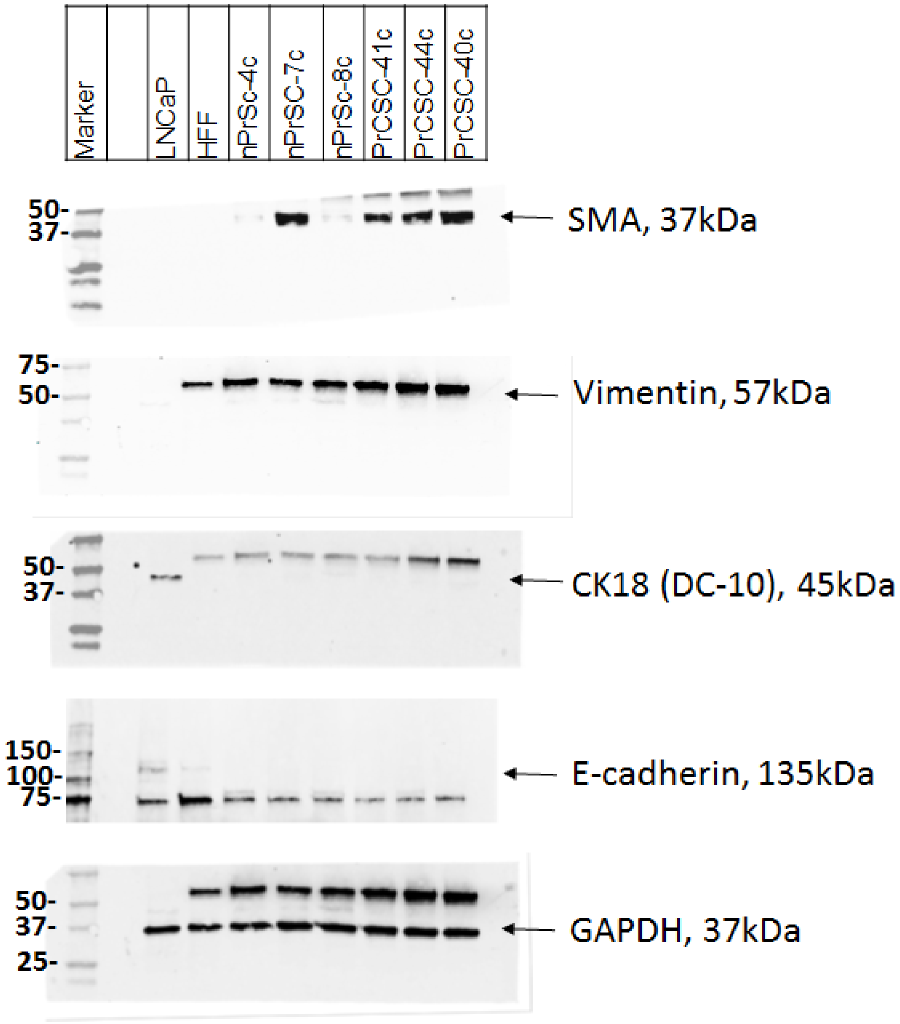

Supplementary Figure S3. Full unedited gels applied to Figure 5B.
